# Successful ICSI in Mice Using Caput Epididymal Spermatozoa

**DOI:** 10.3389/fcell.2019.00346

**Published:** 2019-12-17

**Authors:** Raúl Fernández-González, Ricardo Laguna, Priscila Ramos-Ibeas, Eva Pericuesta, Víctor Alcalde-Lopez, Serafín Perez-Cerezales, Alfonso Gutierrez-Adan

**Affiliations:** Departamento de Reproducción Animal, INIA, Madrid, Spain

**Keywords:** ICSI, caput, epididymis, mice, sperm

## Abstract

Spermatozoa undergo their last phase of spermiogenesis, known as maturation, as they pass through the epididymis. A recent report indicates that mouse immature spermatozoa retrieved from the caput epididymis for intracytoplasmic sperm injection (ICSI) give rise to embryos with multiple developmental defects. Further, these embryos were unable to develop to term after their transfer to surrogate mothers. Herein, we examined the potential of mouse caput spermatozoa to produce normal embryos by comparing the use of caput vs. cauda epididymal spermatozoa for *in vitro* fertilization (IVF) or ICSI. Two methods for the separation of sperm heads prior to ICSI were also compared: freezing/thawing or drawing through a syringe. We found that in contrast to caudal spermatozoa, caput spermatozoa failed to produce embryos via IVF, confirming their immature state. However, regardless of the method employed for the separation of sperm heads, similar efficiencies of blastocyst production *in vitro* and development to term after transfer to surrogate mothers were observed following ICSI using both caput and cauda epididymal spermatozoa. It therefore seems that mice spermatozoa recovered from the caput epididymis are as valid as caudal spermatozoa for the production of embryos and offspring by ICSI.

## Introduction

Spermatozoa acquire their final mature state during passage through the epididymis. Mammalian immature sperm produced in the testis move from the ducts of the testis into the caput epididymis, where they are completely immobile and unable to fertilize an egg cell; then they are transported through the epididymis to the cauda region as a result of rhythmic contractions of this organ, where they undergo complex modifications, acquire motility and become fertilization-competent, denominating then mature sperm (Gervasi and Visconti, 2017). Thus, in theory, ejaculated sperm should have a better fertilizing capacity than sperm recovered from the testis as they have transited through the epididymis, where the final steps of maturation take place. These steps include compaction of sperm chromatin, formation of disulfide bridges between protamines and epigenetic remodeling (Ariel et al., 1994; Haidl et al., 1994; Perez-Cerezales et al., 2012). Chromatin maturation is required to fully stabilize and protect DNA from damage induced by endogenous nucleases or reactive oxygen species during sperm passage through the epididymis (Irvine et al., 2000; Perez-Cerezales et al., 2012). The importance of sperm maturation in the epididymis has also been confirmed by *in vitro* fertilization (IVF) assays performed with spermatozoa collected from different sites of the epididymis. In these assays, sperm retrieved from the distal part acquire a higher motility and ability to fertilize the oocyte (Sullivan and Mieusset, 2016). The epididymis is also able to eliminate defective or immature germ cells (Ramos-Ibeas et al., 2013).

Recently, it has been reported that small RNAs are trafficked to mammalian sperm during the process of post-testicular maturation in the epididymis (Sharma et al., 2018). It has been also described that 2-cell embryos generated via intracytoplasmic sperm injection (ICSI) using sperm obtained from the proximal (caput) versus distal (cauda) epididymis show similar RNA expression, but as development progresses, their gene expression profiles diverge (Conine et al., 2018). Further, embryos arising from caput spermatozoa (caput embryos) have been shown to have multiple developmental defects, and when transferred to surrogate mothers were unable to reach term (Conine et al., 2018). However, the injection of cauda- specific microRNAs (miRNAs) into caput zygotes was observed to suppress the specific gene expression profile of caput embryos and rescue their potential for development. These results indicate that dynamic small RNAs are essential for sperm maturation and also necessary for successful development and were taken as evidence that sperm-borne small RNAs derived from near the cauda region are critical for fetal development (Conine et al., 2018; Sharma et al., 2018). However, both testis spermatids and sperm support full-term development when injected into oocytes (Kimura and Yanagimachi, 1995; Moreira et al., 2016). In this context, the findings of Conine et al. (2018) suggest that the ability of sperm to support full-term embryo development after ICSI is acquired in the testis, then lost in the caput epididymis, and re-acquired during epididymal transit prior to arriving at the cauda. However, this hypothesis contradicts the results reported by others showing that ICSI produces normal offspring using caput sperm (Suganuma et al., 2005; Zhou et al., 2019).

Our hypothesis is that the developmental potential of caput and cauda fertilized embryo is similar and that the discrepancies of the results reported by Conine et al. (2018) respect to the literature could be due to differences in ICSI protocols, mainly in the technique used to separate the head from the tail of the sperm, since the different techniques can produce different degrees of alterations in the sperm (Tateno, 2008). Mouse spermatozoa are particularly sensitive to mechanical damage (Mazur et al., 2000), and these damages can be more decisive for the sperm of the caput, since it has been shown that there are more DNA fragmentation and less chromatin compaction in the caput than in the cauda epididymal spermatozoa (Perez-Cerezales et al., 2012), and that sperm obtained from the caput are more susceptible to nuclease-dependent DNA damage than sperm obtained from other epididymal regions (Yamauchi et al., 2007). Moreover, it has been reported that ICSI in mice using DNA-fragmented sperm has been linked to altered gene expression at the preimplantation stage, reduction of fetal development, an increased risk of genetic and epigenetic abnormalities both in embryos and offspring (Ramos-Ibeas et al., 2014). Separation of the sperm heads from the tail prior to ICSI is commonly conducted by sonication, by fine needle shearing, by unprotected freezing, by piezo pulses, or by detergent treatment, and all these protocols may produce different level of chromosome aberrations in mouse (Martin et al., 1988; Tateno, 2008).

Our study addresses the reproductive potential of caput spermatozoa used for IVF or ICSI. As a positive control, we used caudal spermatozoa, regularly used for these procedures in mice. Our objective was to test the reported inability of caput spermatozoa to produce viable ICSI-derived mice by injecting into oocytes caput or cauda epididymal sperm recovered from standard B6D2F1 hybrid and inbred B6 mouse strains using similar protocols to previously described (Conine et al., 2018).

## Materials and Methods

### Handling of Mice and Sperm Samples

**FIGURE 1:**
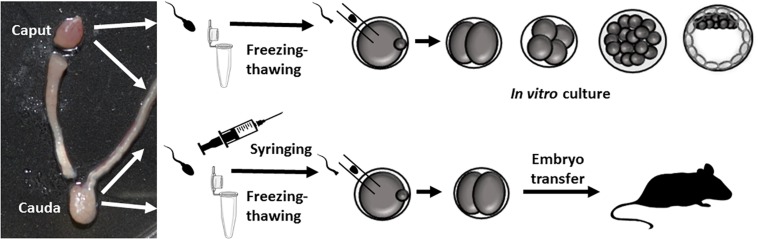
Outline of the experiments carried out. Epididymis dissections showing the caput and cauda regions used. Paired caput and cauda sperm samples (from the same animal) were injected into control oocytes, and 2-cell embryos were *in vitro* cultured or transferred to pseudo-pregnant females.

Experiments in mice were carried out in strict accordance with recommendations of the guidelines of the European Community Council Directive 86/609/EEC. The study protocol was approved by the Committee on the Ethics of Animal Experiments of the INIA (2016 permit number CEEA 2014/025). Spermatozoa were collected from the caput and caudal epididymis of 20-week-old B6D2F1 males (C57/BL/6JxDBA/2J) and B6 (C57BL/6NHsd) mice (Figure 1). Sperm were released by making an incision in the epididymal tissue and then squeezing to release its contents. The sperm were then suspended in M2 medium (Sigma–Aldrich, Madrid, Spain) for the ICSI experiments, or in human tubal fluid medium (HTF) supplemented with 1% BSA for IVF tests. To capacitate the sperm for the IVF experiments, samples were incubated for 30 min under an atmosphere of 5% CO_2_ at 37°C.

### Intracytoplasmic Sperm Injection (ICSI) and *in vitro* Fertilization (IVF)

For our ICSI experiments, the caput and caudal epididymis of the same animal were dissected separately, and sperm collected in M2 medium (6 adult individuals) as described previously (Perez-Cerezales et al., 2012). The sperm was mixed with five volumes of a 10% solution of polyvinyl-pyrrolidone in M2. ICSI was performed in M2 medium at room temperature (Ramos-Ibeas et al., 2014). Sperm heads were decapitated by the freezing-thawing method (Moreira et al., 2003) or by drawing the sperm repeatedly through a fine-gauge needle (25G) into a 1 mL syringe 20–30 times (Conine et al., 2018). In parallel, oocytes were collected at metaphase II from B6D2F1 8-week-old females, which were superovulated by standard intraperitoneal injection (Moreira et al., 2005), and incubated with hyaluronidase (300 μg/mL) to remove cumulus cells. After allowing their recovery for 15 min, sperm were injected into the oocytes in M2 at room temperature. Surviving zygotes were cultured until the blastocyst stage to check their development or until the 2-cell stage for the embryo transfer experiments.

For the IVF experiments, oocytes were obtained from superovulated female mice as described above. The method used has been described elsewhere (Hourcade et al., 2010). Briefly, 2.5–10.0 μL of fresh caput or cauda epididymal sperm were added to each fertilization drop to achieve a final concentration of ∼1–2 × 10^6^ spermatozoa/mL. Four hours after oocyte and sperm co-incubation at 37°C in a humidified atmosphere of 5% CO_2_ in air, putative zygotes were washed and cultured in KSOM.

Statistical differences between caput- and cauda- derived embryos and offspring were assessed using an unpaired *t*-test and ANOVA (SigmaStat package), respectively. Significance was set at *p* < 0.05.

## Results

Sperm from the cauda and caput epididymis were collected from six 20-week-old sexually mature B6D2F1 and four inbred B6 male mice (Perez-Cerezales et al., 2012) and prepared according to requirements for IVF or ICSI (Figure 1). For IVF, the sperm were capacitated for 30 min and for ICSI the sperm were frozen without a cryoprotectant (Moreira et al., 2003) or drawn repeatedly through a fine-gauge needle into a 1 mL syringe 20–30 times (Conine et al., 2018) to obtain tail-free sperm heads. Our IVF experiments revealed that only caudal sperm had the capacity to fertilize oocytes. Thus, using caudal sperm, 96.29 ± 3.70% of fertilized oocytes (*n* = 48) underwent 2-cell cleavage and all of these embryos developed to blastocysts. In contrast, no fertilization was produced with sperm obtained from the caput epididymis: only 4 out of 128 (3.25 ± 3.25%) fertilized oocytes developed to the 2-cell stage, and none developed into blastocysts. This result could be explained by a lack of motility of caput sperm.

**FIGURE 2:**
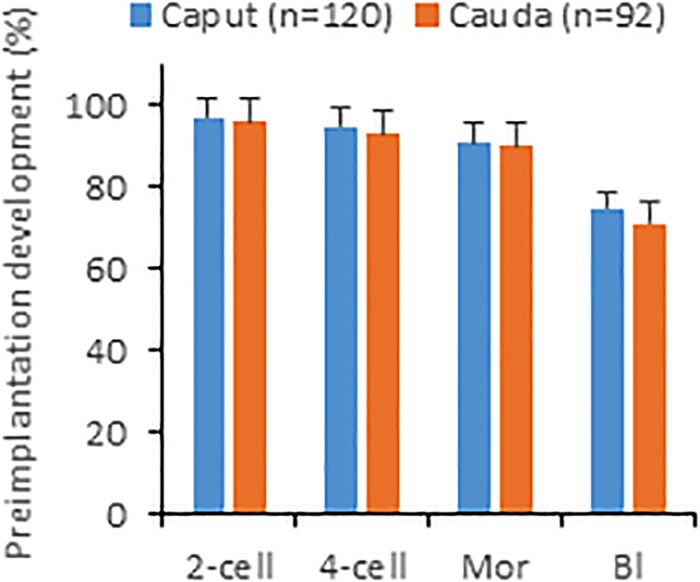
*In vitro* development of embryos produced by injecting sperm from caput or cauda epididymis into oocytes. Percentages shown are calculated with respect to oocytes surviving injection. Mor, morulae; Bl, blastocyst. Values are mean ± SEM. No significant differences between groups were detected (unpaired *t*-test, *p* ≤ 0.05).

We next microinjected caput and caudal sperm (heads decapitated by freezing-thawing) of B6D2 mice into oocytes and found that embryos produced with both sperm types developed *in vitro* to the blastocyst stage at similar efficiencies (Figure 2). Given that caput-derived embryos were perfectly capable of developing to the blastocyst stage *in vitro* (57% of blastocysts produced by caput sperm vs. 63% by caudal sperm, four replicates and more than 100 embryos produced in each group), we then generated both caput- and cauda- derived embryos by ICSI using paired sperm samples from the same animal, and surgically transferred 15–20 2-cell embryos into pseudopregnant surrogate mothers to analyze their *in vivo* developmental capacity. First, we used our classic method of fast freezing/thawing to separate sperm heads. As we show in Table 1, we found similar percentages of cleaved embryos and live offspring using caput or caudal sperm. We then used the drawing through a syringe method to decapitate sperm (Conine et al., 2018), and using both B6D2 and B6 mice, we also observed similar percentages of 2-cell embryos and live offspring derived from caput or caudal sperm. Interestingly, we noted a small but non-significant reduction in the number of live offspring when sperm were treated with the syringe, and also a non-significant reduction in the percentage of live offspring generated from caput sperm. In total, 20 and 30 pups were obtained by both procedures from caput and cauda sperm, respectively. Birth weights and blastocyst development were similar between the caput and cauda groups and both males and females were fertile (*n* = 8 per group).

**TABLE 1:** Development of oocytes injected with sperm from caput vs. caudal epididymis.

**Sperm source**	**Mouse strain**	**Sperm treatment**	**Oocytes surviving injection (%)**	**2-cell embryos at 24 h (% surviving)**	**No. embryos transferred (no. recipients)^*a*^**	**No. live offspring (% implantations)**
Caput	B6D2	Freezing/thawing	167/192 (86.9)	134 (80.2)	134 (5)	15 (11.2)
Cauda	B6D2	Freezing/thawing	90/116 (77.6)	78 (86.7)	78 (4)	19 (24.4)
Caput	B6D2	DWG T syringe	46/53 (86.8)	35 (76.1)	35 (2)	3 (8.6)
Cauda	B6D2	DWG T syringe	44/60 (73.3)	36 (81.8)	36 (3)	6 (16.7)
Caput	B6	DWG T syringe	39/50 (78)	32 (76.9)	32 (2)	2 (6.3)
Cauda	B6	DWG T syringe	38/50 (76)	31 (81.6)	31 (2)	5 (16.7)

*Four independent experiments were conducted. B6D2 = C57/BL/6 JxDBA/2 J; B6 = C57BL/6NHsd; DWG T syringe = Drawing through syringe. ^*a*^All recipients were pregnant. *P* > 0.05 Chi-square analysis.*

## Discussion

A recent report describes that embryos generated via ICSI using immature sperm derived from the caput epididymis show multiple defects during the peri-implantation period and quickly fail to undergo implantation (Conine et al., 2018). The authors of this paper also observed that these defects could be rescued by the injection of small RNAs derived from extracellular vesicles (epididymosomes), which deliver their small RNA repertoire to mature sperm, suggesting that small RNAs in mature sperm play major roles during early mouse embryogenesis. Here, we report that similar embryo development and offspring production results for ICSI using immature sperm from the caput epididymis or mature sperm from the caudal epididymis.

In mice, ICSI is performed with isolated sperm heads. However, sperm heads can be obtained by different protocols and this could have an appreciable impact on the sperm. In our hands, both protocols used to generate individual sperm heads (freezing/thawing, the standard method used in our laboratory, and drawing through a syringe, the method employed by Conine et al. (2018) produced healthy offspring from immature caput sperm. However, the number of live offspring generated with caput sperm was slightly lower than that generated with caudal sperm. In effect, this could be due to differences in maturation and sperm chromatin status between caput and caudal sperm. We earlier reported that the immaturity of mouse spermatozoa in the caput epididymis was revealed by a higher permeability of membranes to propidium iodide (PI) (Perez-Cerezales et al., 2012). Sperm chromatin undergoes the formation of disulfide bridges between protamines during the transit through the epididymis, increasing compaction of the genetic material (Golan et al., 1996). Also, using the comet assay and sperm chromatin structure assay (SCSA), we previously described different maturation stages of spermatozoa along the epididymis consisting of more DNA fragmentation and less chromatin compaction in the caput- than cauda epididymal spermatozoa (Perez-Cerezales et al., 2012). These differences in chromatin structure could make caput sperm more sensitive to decapitation procedures. Perhaps, a more aggressive technique will lead to more severe chromatin alterations in spermatozoa from the caput epididymis, or leave DNA more exposed and thus susceptible to degradation by endogenous or exogenous nucleases. Oocytes can support the preimplantation development of sperm with damaged chromatin, but major developmental defects appear later, after implantation (Fernandez-Gonzalez et al., 2008). This is in line with clinical observations that ejaculated sperm with damaged DNA go through normal preimplantation embryonic stages but fail to develop beyond this stage (Tesarik et al., 2004). Further, another two procedures designed to separate the sperm head from tail have been used to produce normal offspring from caput sperm (Suganuma et al., 2005; Zhou et al., 2019). The sperm head can be separated from the midpiece and tail by applying one or a few piezo pulses (Suganuma et al., 2005) or through sonication or the use of detergents like Triton X-100 or 3-[(3-cholamidopropyl) dimethylammonio]-1-propanesulfonate (Zhou et al., 2019).

There are several methodological differences between Conine et al. (2018) and previous studies (Suganuma et al., 2005; Zhou et al., 2019) which could potentially account for the discrepancy about the ability of caput-derived embryos to implant. In our experiments we used similar protocols to Conine et al. (2018) with the exception of the mouse strain background (we use B6D2 hybrids and inbred B6, and Conine et al. (2018) use inbred FVB). Further, other inbred mice have been also used, like 129Sc (Suganuma et al., 2005), showing to produce normal caput-derived mice. Although we cannot eliminate the possibility that the ability of caput-derived embryos to implant could be affected by strain background our results rule out the general character of the findings reported by Conine et al. (2018) for the mouse species.

In summary, sperm from the caput region of the epididymis are incapable of IVF but, contrary to recent findings (Conine et al., 2018), we here confirm that healthy fertile mice can be produced by ICSI from caput sperm.

## Data Availability Statement

All datasets generated for this study are included in the article/supplementary material.

## Ethics Statement

The animal study was reviewed and approved by the Committee on the Ethics of Animal Experiments of the INIA (2016 permit number CEEA 2014/025).

## Author Contributions

RF-G and RL produced the ICSI mice and collaborated in the phenotyping. PR-I, EP, VA-L, and SP-C performed all the experiments and co-wrote the manuscript. AG-A conceived the experiments and co-wrote the manuscript.

## Conflict of Interest

The authors declare that the research was conducted in the absence of any commercial or financial relationships that could be construed as a potential conflict of interest.
